# Local reaction kinetics by imaging^☆^

**DOI:** 10.1016/j.susc.2015.05.021

**Published:** 2016-01

**Authors:** Yuri Suchorski, Günther Rupprechter

**Affiliations:** Institute of Materials Chemistry, Vienna University of Technology, Getreidemarkt 9, 1060 Vienna, Austria

**Keywords:** Photoemission electron microscopy, Catalysis, CO oxidation, Catalytic ignition, Platinum, Palladium

## Abstract

In the present contribution we present an overview of our recent studies using the “kinetics by imaging” approach for CO oxidation on heterogeneous model systems. The method is based on the correlation of the PEEM image intensity with catalytic activity: scaled down to the μm-sized surface regions, such correlation allows simultaneous local kinetic measurements on differently oriented individual domains of a polycrystalline metal-foil, including the construction of local kinetic phase diagrams. This allows spatially- and component-resolved kinetic studies and, e.g., a direct comparison of inherent catalytic properties of Pt(hkl)- and Pd(hkl)-domains or supported μm-sized Pd-powder agglomerates, studies of the local catalytic ignition and the role of defects and grain boundaries in the local reaction kinetics.

## Highlights

• An overview of the recent studies using the “kinetics by imaging” approach for CO oxidation on model systems is presented.
• The approach allows spatially-t-resolved kinetic studies with a spatial resolution of the used PEEM, e.g. on the μm-sized domains of polycrystalline samples.
• CO oxidation on Pd(hkl)-domains and on supported Pd-powder, role of defects and grain boundaries in the reaction kinetics are described as “case studies”.

## Graphical abstract

**Figure:**
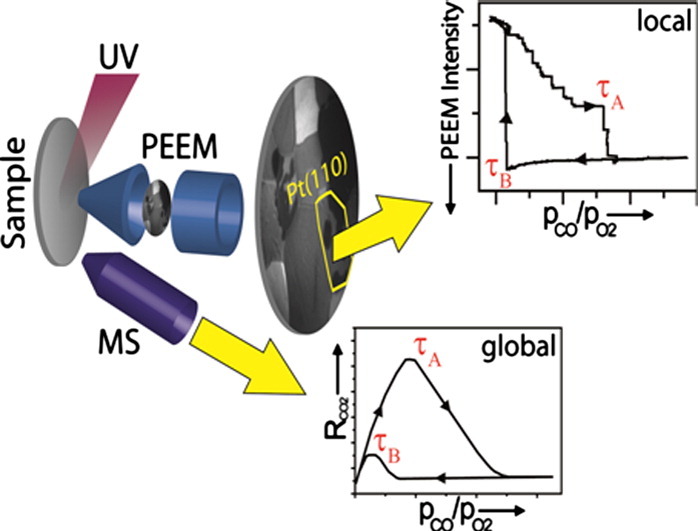

## 1 Introduction

Already 40 years ago in an early work of Menzel and Kraemer it was demonstrated, that one can visually distinguish between the adsorbed CO and oxygen on the transition metal surface imaged by electron emission microscopy: different emission intensities were observed for the CO_ad_ and O_ad_ layers on Ru during the FEM (field electron microscopy) imaging [1]. Physical reason for this lies in the differing work function values of the CO- and oxygen-covered Ru surface (FEM is mainly a “work function” microscope [2]). Twenty years after these observations, a straight-forward correlation between the work function of the CO/O-covered surface and its catalytic activity in CO oxidation on Pt was experimentally established [3]. Thus it could be expected, that generally the image intensity in the “work function based” electron emission microscopes such as e.g. PEEM (photoemission electron microscopy), can provide information about the catalytic activity of the imaged surface, at least in CO oxidation. This relation was tacitly assumed in numerous PEEM observations of the CO oxidation on Pt-metal surfaces and also used for qualitative considerations [3], [4], [5], [6]. The above studies were performed mainly for the single crystal surfaces (where the local activity corresponds to the averaged one), therefore the mass spectrometric (MS) measurements running parallel to PEEM observations, provided sufficient information about the reaction kinetics [4], [5]. In the case of the spatially-heterogeneous model systems, the MS approach meets, due to the data-averaging principle, its limits: MS does not distinguish between product species originating from different regions of the sample. Simplified model systems with active regions separated by sub-mm distances may allow for the use of a “scanning-MS” with gas sampling via a capillary leak [7]. Nevertheless, as for every scanning procedure, parallel measurements for many different locations are not possible with this method. Additionally, the products from neighboring locations might also attain the sampling nozzle-sniffer.

In turn, the correlation of the catalytic activity with the local work function opens the way to a “kinetics by imaging” approach where the information can be obtained solely from the work function dependent image intensity. Since such correlation must be valid also for the microscopically small areas of the surface, such local information can be obtained down to the resolution limit of the corresponding microscope. For PEEM this may allow spatially-resolved studies of local kinetic instabilities such as e.g. kinetic phase transitions on the μm- to tens of nm scale for the newest devices.

In the present contribution we present an overview of our recent studies using the “kinetics by imaging” approach for CO oxidation on heterogeneous model systems such as individual domains of polycrystalline Pt and Pd foils or Pd powder agglomerates supported by Pt foils.

## 2 Basics and experimental

**Fig. 1:**
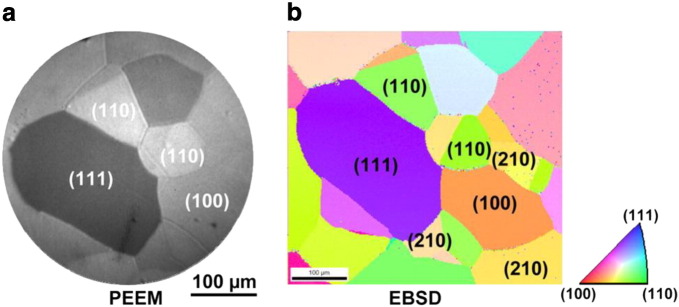
An example of a polycrystalline Pt foil: a) as imaged in PEEM; and b) identification of individual surface domains by EBSD. Note the inverse pole figure assigning the corresponding directions. From [13].

As already mentioned above, the idea of the approach is based on the fact that the photoemission yield is directly dependent, via work function, on the adsorbate coverage. For the CO oxidation, it means that a CO or oxygen adlayer can be unambiguously identified from the PEEM image and, after the corresponding calibration, even the CO or oxygen surface density can be directly extracted from the PEEM image intensity [8]. Since the CO or oxygen coverage governs the rate of CO_2_ formation the PEEM image intensity may serve as an indicator for the reaction rate. The CO oxidation on Pt-metals was intensively studied by PEEM since 1990s [3], [4], [5], [6], including the observation of global kinetic transitions occurring on the single-crystal surfaces, such as Pt(111) [9], but the next logical step exploiting the parallel imaging principle of PEEM and the validity of the “catalytic activity – image intensity” correlation also for small areas, was not performed till 2010 [10]. The first studies of local kinetics using PEEM imaging were performed for polycrystalline Pt and Pd foils consisting on μm-sized grains forming a surface which exhibits differently oriented domains of corresponding size [10], [11], [12], [13]. Fig. 1 shows an example of such a polycrystalline foil including the electron backscatter diffraction (EBSD) analysis of crystallographic orientation of particular domains. During the catalytic experiments, all the domains of the sample are exposed to the exactly identical conditions and information is collected, due to the parallel imaging principle of PEEM, simultaneously for all domains, providing thus perfect options for a comparison of the behavior of different orientations.

The experiments were performed in a UHV system consisting of two independently operated chambers connected with each other by a sample transfer line, thus allowing a common reactive gas atmosphere in the 10^− 4^–10^− 9^ mbar range. The “microscopy” chamber is equipped with a PEEM, (Staib instruments), a quadrupole mass spectrometer (QMS, MKS instruments), a LEED system (Omicron), a high purity gas supply system (O_2_: 99.99%, CO: 99.97%) and sample preparation facilities for cleaning the sample by argon ion sputtering and annealing. The “spectroscopy” chamber is equipped with an XPS-system (Phoibos 100 hemispherical energy analyzer and XR 50 twin anode X-ray source, SPECS). The details of the experimental setup are described elsewhere [10], [11], [12], [13].

**Fig. 2:**
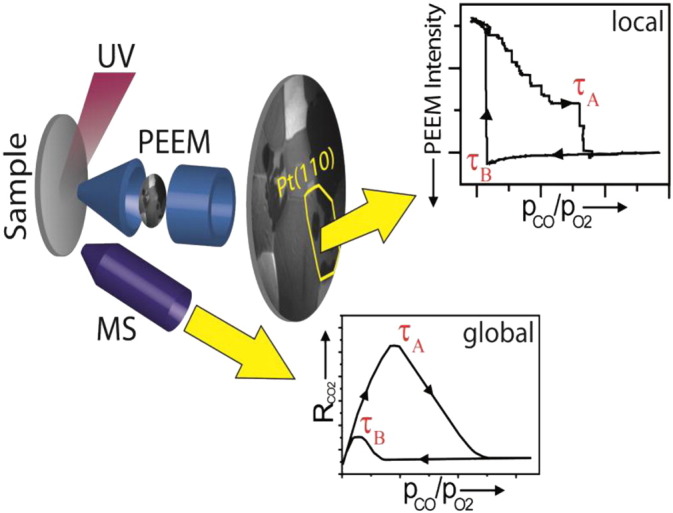
Principle of the experiment. The ongoing catalytic reaction is simultaneously monitored by PEEM and MS. Global (averaged) MS data (lower inset) can be correlated with the spatially resolved data resulting from the intensity analysis of the video-PEEM images (upper inset). From [12].

The scheme of measurements is shown in Fig. 2: the global CO_2_ formation rate in the CO oxidation reaction, originating from the whole sample is monitored by QMS yielding the *global* reaction kinetics. Simultaneously, PEEM is applied to visualize the reaction *in situ*. The contrast of a PEEM image which is formed by photoelectrons emitted from the surface upon UV light illumination is attributed to the local work function variations across the sample. These variations are sufficient to monitor the CO oxidation reaction. The digital analysis of the recorded video sequences allows to follow the reaction on the individual grains of the polycrystalline sample and to obtain *local* kinetic transitions on specific domains or different ingredients, in the case of spatially separated active components.

## 3. Applications

**Fig. 3:**
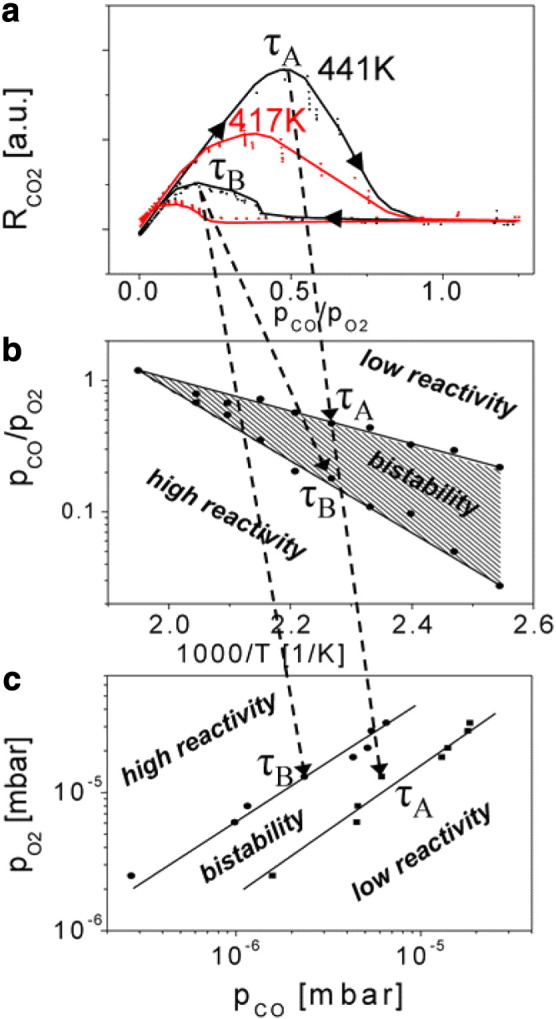
Global mass spectroscopy (MS) studies of CO oxidation on polycrystalline Pt foil: (a) hysteresis of the CO_2_ production rate at cyclic variation of the CO pressure at an O_2_ pressure of 1.3 × 10^− 5^ mbar and a temperature of 417 K (red line) and 441 K (black line). The τ_A_ and τ_B_ values obtained from the hysteresis curves in (a) are used for the phase diagrams in Fig. 3b and c as indicated by the arrows. (b) Global kinetic phase diagram at constant oxygen pressure of 1.3 × 10^− 5^ mbar for CO oxidation on polycrystalline Pt foil, as obtained by *in situ* MS. (c) Corresponding kinetic phase diagram at constant temperature of 441 K for CO oxidation on polycrystalline Pt foil as obtained by *in situ* MS. From [12].

Our particular interest was directed to kinetic instabilities in CO oxidation, such as catalytic ignition and extinction which occur under unsteady-state conditions and are important for catalytic treatment of automotive exhaust gases [14], [15]. A specific applied problem is the so-called “cold-start”, where the effectivity of the catalytic converter remains very low until it warms up to the “ignition” temperature, at which the reaction rate rapidly switches from low to high conversion. Any method aiming an accelerated heating of the converter in order to quickly reach the critical temperature, such as operation at lean air-to-fuel ratio, exhaust system combustion devices, secondary air injection into the exhaust, electrically heated catalysts, causes additional energetic expenses [16]. Thus there is a substantional interest in studies of ignition–extinction processes which could promise an “energy-neutral” solution via lowering the critical temperature. In the case of CO oxidation, the sudden increase of the reaction rate at increasing temperature is related to the “kinetic phase transition” occurring when the CO covered (poisoned) surface becomes more or less abruptly oxygen covered (catalytically active) as a result of kinetic unbalance in the competitive coadsorption of CO and oxygen at increasing temperature. The term “kinetic phase transition” results from the similarity between the non-equilibrium kinetic transitions occurring in the reaction and equilibrium phase transitions: as already noticed by Schlögl in the 1970s, in both equilibrium and non-equilibrium phase transitions a crucial role is played by cooperative phenomena forming, for example, ordered structures in an equilibrium, and self-organizing dissipative structures in a non-equilibrium situation [17], [18]. Lots of experimental studies were performed for CO oxidation, where the kinetic transitions τ_A_ (see Fig. 3a) from high reactivity (oxygen covered surface) to low reactivity (CO poisoned surface) and vice versa (τ_B_) were obtained by MS tracking the hysteresis-like CO_2_ production rate (R_CO2_ curve) at varying e.g. p_CO_ at constant p_O2_ and T [9], [19], [20]. Fig. 3 shows a typical example of such classical MS study for a polycrystalline Pt foil: by measuring series of such hysteresis curves at different temperatures (Fig. 3a), so-called *kinetic phase diagram* can be constructed where the regions of high and low reactivity and bistability can be indicated (Fig. 3b,c). Such diagrams are extremely helpful for revealing of promoter-, and surface-modification effects [13], [19], [20], [21].

The PEEM based approach described above, provides the possibility for similar studies but in a spatially-resolved way: transition points τ_A_ and τ_B_ can be obtained locally for individual regions of the sample under exactly the same conditions. This is particularly interesting for catalytic ignition studies: the comparison between different samples needs not only an exact keeping of constant parameters such as reactants pressure in the recurring experiments, but also exact repetition of the temperature ramps. Both conditions are provided automatically in the present approach.

**Fig. 4:**
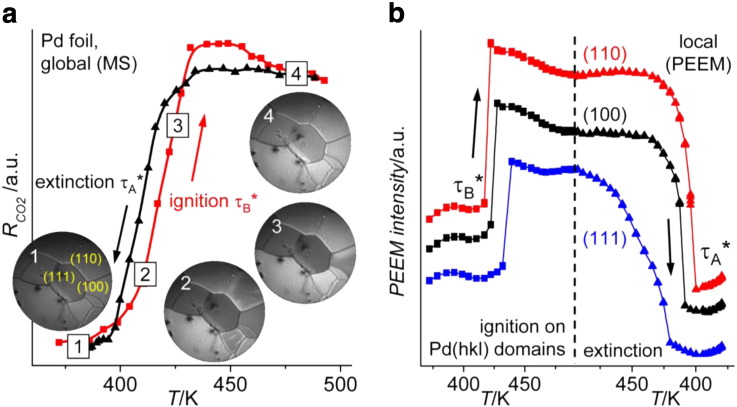
Global (MS) versus local (spatially-resolved, PEEM) monitoring of catalytic ignition: a) global MS measurements of ignition (red squares) and extinction curves (black triangles) on polycrystalline Pd foil. CO_2_ production rate measured by MS during cyclic variation of the sample temperature (rate: 0.5 K s^− 1^) at constant *p*_CO_ = 5.8 × 10^− 6^ mbar and *p*_O2_ = 1.3 × 10^− 5^ mbar is plotted versus sample temperature. Simultaneously recorded PEEM video-sequences illustrate the ignition process: Frame (1) inactive, CO covered surface; Frame (2)—ignition begins on (110) domains; Frame (3)—ignition continues on (100) domains; Frame (4)—oxygen covered, active surface. b) Laterally resolved ignition/extinction measurements: local PEEM intensity for the individual (110), (100), and (111) domains during the same cyclic temperature scan as in (b). The vertical dashed line indicates the turning point from heating to cooling. From [11].

Fig. 4 shows the results of such spatially-resolved study of the catalytic ignition in individual low-index domains of a polycrystalline Pd foil [11].

Following the temperature ramp from 372 K to 493 K (rate of 0.5 K/s) at constant p_CO_ = 5.8 × 10^− 6^ mbar and p_O2_ = 1.3 × 10^− 5^ mbar, the global CO_2_ rate suddenly increases indicating the transition τ_B_* from the state of low catalytic activity (CO-poisoned surface; video-frame 1 in Fig. 4b, dark contrast) to the state of high catalytic activity at which the surface becomes oxygen covered (frame 4; bright contrast). Analogous to the MS signal in the overall CO_2_ reaction rate, the jumps in the local PEEM intensity represent the local kinetic transitions on the individual grains (Fig. 4b). These transitions do not occur simultaneously on the different orientations but show a pronounced structure sensitivity with clearly identifiable critical temperatures of 417 K for Pd(110), 423 K for Pd(100) and 432 K for Pd(111). A similar observation was made for the reaction extinction, i.e. for the transition τ_A_* from the high reactivity to the low reactivity state upon cooling the sample. Again, the curve of the *global* CO_2_ production rate appears to be “smoothened out” (black curve in Fig. 4a), whereas *local* extinction on the individual grains occurs rather sharply and independently from each other (Fig. 4b). This clearly shows the limitation of averaging techniques such as mass-spectroscopy, which can not reveal the important local kinetics.

**Fig. 5:**
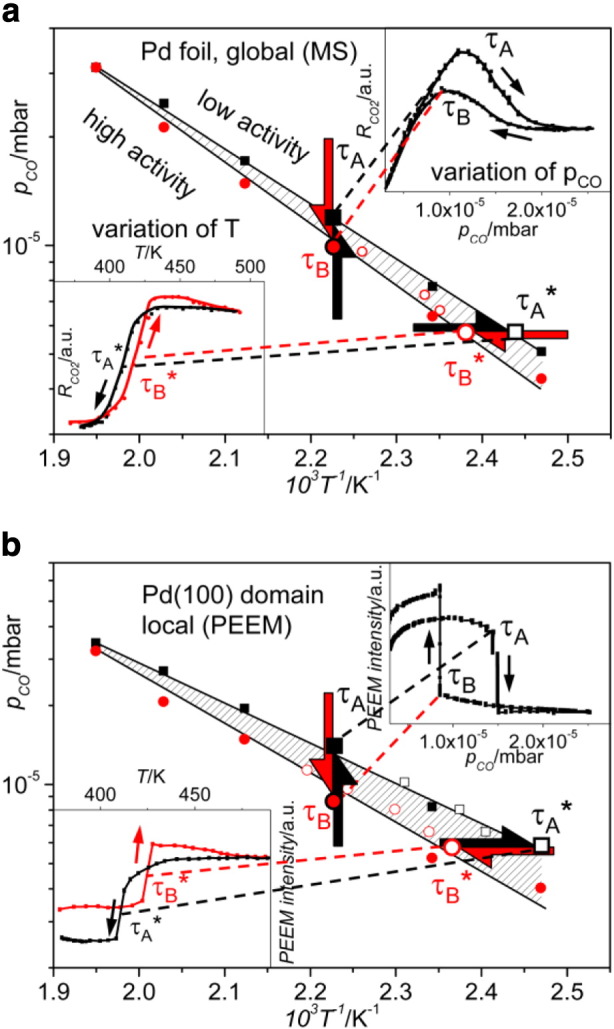
Ignition/extinction measurements versus pressure variations: global (a) and local (b) kinetic phase diagram illustrating the CO oxidation reaction on polycrystalline Pd foil (a) and on a single Pd(100) domain of the Pd foil (b). Note the agreement of the transition points τ_A_* and τ_B_* obtained at varying T (from the ignition/extinction curves shown in the left insets) with the diagram obtained via cyclic variation of p_CO_ (from the poisoning/reactivation curves in the right insets). The dashed regions indicate the range of bistability. From [11].

Such *isobaric* experiments are usually performed in technical catalytic studies, the typical *surface science* approach is based rather on isothermal measurements as shown in Fig. 3. Fig. 5 links these two approaches comparing directly the isothermal and isobaric data, for both global and spatially resolved measurements: ignition/extinction measurements (lower inset in Fig. 5a) provide isobaric transition points τ_A_* and τ_B_*, in turn variation of p_CO_ at constant temperature (upper inset in Fig. 5a) deliver τ_A_ and τ_B_ values.

Isothermal and isobaric sets of data lead, as appears from Fig. 5a,b, to identical kinetic phase diagrams, both for the global MS and for the local PEEM measurements, i.e. the isothermal monitoring of kinetic transitions yields the same results as the common technical catalysis approach.

**Fig. 6:**
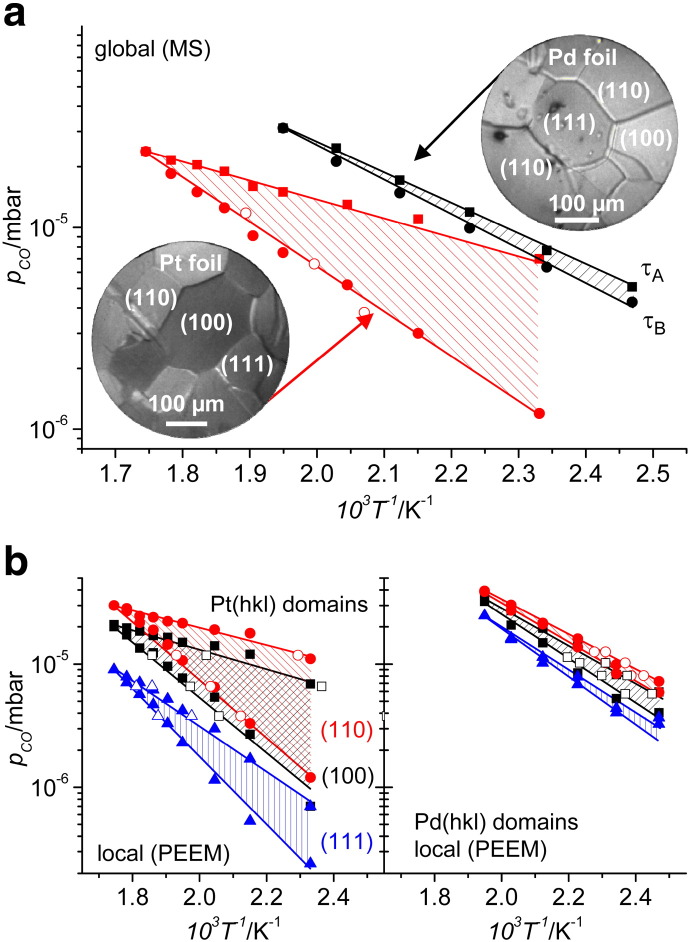
Palladium versus platinum in CO oxidation. a) Comparison of the global kinetic phase diagrams (by MS) at constant oxygen pressure (p_O2_ = 1.3 × 10^− 5^ mbar) of polycrystalline Pt (filled red squares and circles) and Pd (black squares and circles). Open circles are ignition points for Pt. b) Corresponding local kinetic phase diagrams for individual Pt(hkl) domains (left) and Pd (right), obtained by local PEEM intensity analysis. Open symbols are the local ignition and extinction points. From [11].

PEEM monitoring of kinetic transitions appears to be not only well suitable for effective comparison of reaction behavior of different crystallographic orientations of the same metal but also of different samples, e.g. Pt and Pd. Fig. 6 shows such a comparison, where global reaction behavior of Pt and Pd foil is compared (Fig. 6a) as well as individual (hkl) domains are confronted (Fig. 6b). It is clearly visible that the global and the local kinetic phase diagrams of Pd foil are situated at significantly higher CO partial pressure and the bistability range is much narrower for Pd than for Pt foil. In particular, this means that for Pd the transition τ_A_ from the high to the low reactivity state occurs at higher CO partial pressure than for Pt, and that the reverse transition t_B_ also occurs at a higher CO-to-oxygen ratio than for Pt. In other words, Pd is the better (more CO-tolerant) catalyst than Pt under the current conditions because *more* CO is needed to poison the Pd surface and a *lower* oxygen-to-CO ratio is sufficient to “reactivate” the Pd surface. Besides that, the bistability regime of Pd disappears at a lower temperature than in the case of Pt, namely at T_Pd_ = 513 K in contrast to T_Pt_ = 573 K, so already at lower temperature Pd cannot be poisoned by CO anymore.

**Fig. 7:**
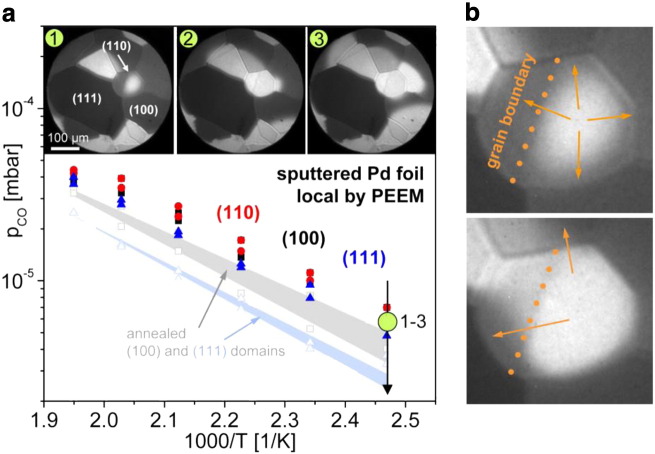
CO oxidation on individual (hkl) domains of a sputtered Pd foil in comparison with the smooth surface. (a) Local kinetic phase diagrams of the individual Pd(110) — (red), Pd(100) — (black) and Pd(111) — (blue) domains of an additionally sputtered Pd foil, in comparison with the (100) and (111) domain of an annealed Pd foil, at constant p_O2_ = 1.3 × 10^− 5^ mbar and different constant temperatures. The local kinetic phase diagrams of the sputtered surface are shifted together and towards higher CO pressures, as compared to the local kinetic phase diagrams of the annealed sample (see Fig. 5a). Frames 1–3: transition τ_B_ at 405 K on the additionally sputtered Pd foil, p_CO_ = 5.9 × 10^− 5^ mbar. (b) The reaction fronts propagate across the grain boundaries. The independency of the individual domains is lifted. From [13].

Additionally to such comparative studies, the imaging approach can reveal the role of such effects as defects of different density, atomic steps, grain boundaries, the role of substrate in supported model catalysts, etc. We have studied the role of defects on an artificially defected Pd surface: a polycrystalline Pd foil was Ar^+^ sputtered, annealed, XPS-proven and then additionally gently Ar^+^ sputtered (E_kin_ = 1 kV, ion dose 2 × 10^16^ cm^− 2^, sample at room temperature) to create defects. The density of resulting defects was controlled by STM [13]. CO oxidation reaction on such a surface was PEEM monitored and compared with the data obtained for the same, but smooth (annealed) surface. The results of such study are summarized in Fig. 7, where the kinetic phase diagrams for individual (hkl) domains of such artificially defected Pd surface and for the same but annealed surface are shown [13].

A significant shift towards higher CO pressure, as compared to that of the smooth surface is observed for the surface modified by additional Ar^+^ sputtering. This indicates that CO oxidation on the sputtered Pd foil is inhibited by CO poisoning only at a considerably higher CO partial pressure and the sputtered surface is also reactivated at a higher CO pressure than the annealed Pd foil. In other words, the defect-rich sputtered sample is much more tolerant towards CO poisoning than the smooth surface. To explain these observations, STM was applied to image both a smooth and an additionally sputtered Pd(111) surface. The STM reveals formation of three-dimensional islands, with a very high density of steps and edges upon sputtering. The uppermost terraces of the mounds have diameters of 2–3 nm, and the slope angles are between 10 and 20°, corresponding to terrace widths of 0.6 to 1.2 nm. This means that about one quarter of all surface Pd atoms are step or kink atoms [13]. It is known that the binding energy of oxygen is considerably higher at low-coordination defect sites than on flat terraces of Pd, i.e. atomic oxygen binds more strongly to a defect-rich Pd surface [22], [23]. Although the CO binding energy is also altered on such defect sites on Pd surface, the impact on the CO adsorption kinetics appears to be rather small when compared to oxygen [24], [25]. Since the energetics governs the kinetics of the competitive CO and oxygen coadsorption, this explains why on the sputtered Pd surface higher CO pressure is required to poison the surface. The higher CO pressure values at which reactivation of the sputtered Pd surface occurs, i.e. the reverse transition τ_B_, can be directly explained by the adsorption kinetics rather than the energetics: due to the high step and defect density, more adsorption sites for oxygen adsorption on a mainly CO-covered surface are available, the sticking probability for oxygen adsorption is considerably higher and therefore, the reactivation occurs at a higher CO-to-oxygen pressure ratio than on the smooth surface.

The examples shown above demonstrate the abilities of the approach for the different domains of the same sample, it is clear however, that such practice can be applied to the multicomponent systems consisting of mixed metal oxides and different supported precious metals, such as Pt, Pd, and Rh. The resulting reaction kinetics is expected to appear quite complex due to the contributions of different individual catalytically active components and their complex interactions with the oxidic substrate [26], [27]. Again, as in the case of differently oriented domains, the usual mass-spectrometric detection of reaction products can hardly solve this task, since it does not distinguish between product species originating from different catalytically active surface sites. To extend the kinetic by imaging approach to the multicomponent systems, a model system consisting on μm-sized Pd powder agglomerates supported by a polycrystalline Pt foil was prepared. Such a model system is well suitable for proving the possibility to study the reaction kinetics not only in a spatially-resolved but also in a component-distinctive way since the parameter ranges of catalytic activity for CO oxidation differ significantly for Pd and Pt [11]. In addition, the work function changes caused by CO and oxygen adsorption on Pt and Pd surfaces lead to differing contrasts in PEEM during the CO oxidation: an oxygen covered Pt surface appears as “dark” [10], [11] in contrary to the Pd surface [11], [13] which appears as “bright” upon oxygen adsorption. This allows an easy differentiation between the Pt support and Pd powder particles down to the resolution limit of the available PEEM (a few μm in our case).

**Fig. 8:**
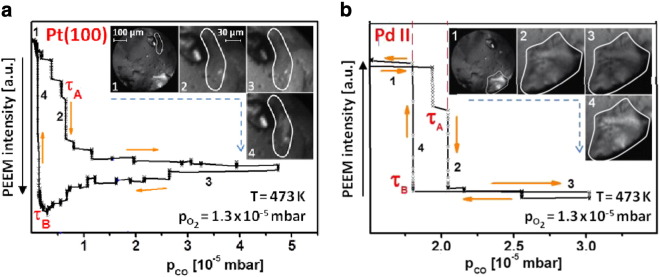
Comparison of the catalytic behavior of a Pt(100) domain of supporting Pt foil and of a Pd agglomerate on the same foil: a) hysteresis-like PEEM intensity plots measured locally for the Pt(100) domain during cyclic variation of the CO pressure at constant temperature of 473 K and a constant partial oxygen pressure of 1.3 × 10^− 5^ mbar. b) the same, but for Pd agglomerate. The insets show chosen PEEM frames for particular characteristic parts of the hysteresis loop. Note the significantly different ranges of bistability (CO pressure values between the τ_A_ and τ_B_ for Pt domain and Pd agglomerate). From [28].

**Fig. 9:**
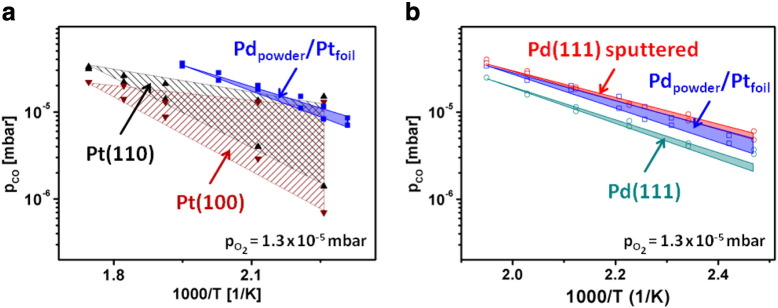
Component-specific kinetic phase diagrams: a) diagrams for Pt(100) and Pt(110) domains of the supporting Pt foil from in comparison with the diagram for the supported Pd agglomerates; and b) the same diagram as in (a) for the supported Pd agglomerates in comparison with diagrams for the ordered Pd(111) surface and sputtered (highly defected) Pd(111) surface (the latter two adapted from [13]). From [28].

Fig. 8 shows an example of such PEEM study: the hysteresis behavior in CO oxidation is monitored for a Pt_foil_/Pd_powder_ sample and the local PEEM intensity is evaluated separately for a Pt(100) domain of the supporting Pt foil and for a μm-sized Pd agglomerate supported by this foil [28]. From the hysteresis loops such as shown in Fig. 8a and measured for different temperatures, kinetic phase diagrams can be constructed for any particular part of the sample within the field of view. Such diagrams for the Pt(100) domain visible in Fig. 8 and for the adjacent Pt(110) domain are shown in Fig. 9a.

Similarly, as in the case of the supporting Pt foil, the local kinetic phase diagrams for the Pd agglomerates can be extracted from a series of local hysteresis curves at different reaction temperatures, for which the same PEEM video-sequences could be used which were exploited for construction of diagrams for Pt(100) and Pt(110) domains.

The common kinetic phase diagram for two Pd powder agglomerates, which behave identically is also plotted in Fig. 9a. It should be noted that all three diagrams in Fig. 9a are obtained in one experiment, i.e. at fully identical conditions and after the identical preparation procedure for all regions of the sample. The diagrams for Pt(100) and Pt(110) are in quantitative agreement with those measured for the corresponding Pt domains without Pd agglomerates [11] and with data for corresponding single crystal surfaces [4]. This means that also for a combined Pd_powder_/Pt_foil_ sample the individual domains of the supporting Pf foil behave independently from each other and from the Pd agglomerates.

It is interesting to compare the results for the Pd powder agglomerates with known data for different Pd surfaces. Fig. 9b shows such a comparison of the data for Pd powder from Fig. 9a with the Pd(111) surface [11], and with the same Pd(111) surface but after Ar^+^ ion bombardement, i.e. with an artificially defected surface [13]. The comparison shows that the reactive behavior of the Pd agglomerates differs significantly from that of the smooth ordered Pd(111) domain, with the CO poisoning (upper boundary of the bistability region) occurring for Pd powder at a considerably higher CO partial pressure than for the smooth Pt(111) surface. Correspondingly, the Pd powder surface is also reactivated at a higher CO pressure than the ordered Pd(111) surface, i.e. the defect-rich Pd powder surface is much more tolerant towards CO poisoning than the smooth ordered surface.

The same effect is observed for the defect-rich Ar^+^ sputtered Pd surface: the diagrams for the Pd powder and for the Ar^+^ sputtered Pd(111) overlap in the Fig. 9b. Apparently, the degree of the surface disorder and the density of defects created by Ar^+^ sputtering is similar to that existing on the surface of Pd agglomerates in the present study. As already noted, the ion bombardment of the Pd surface leads to formation of three-dimensional islands with a very high density of steps and edges [13]. One can imagine that present Pd powder agglomerates have a similarly high density of steps and edges with a considerably higher binding energy of oxygen than that of flat terraces. Similarly, as for the sputtered Pd surface, the impact of defects on the oxygen adsorption prevails over that of CO adsorption. Since the kinetics of the competitive CO and oxygen coadsorption is governed by the energetics of adsorption, this explains why the Pd powder surface can withstand a higher CO pressure prior to being poisoned. The reverse transition τ_B_ occurs also at a higher CO-to-oxygen pressure ratio than on the smooth Pd: due to the high step and defect density of the powder (and sputtered) surface more favorable sites for oxygen adsorption are available, even on a mainly CO-covered surface, i.e. the higher sticking probability for oxygen adsorption forces an “earlier” reactivation.

## 4 Summary

By processing video-PEEM images, acquired *in situ* during a catalytic reaction, local kinetic information can be obtained in a parallel way for different regions of the sample under identical reaction conditions. This allows to compare the inherent catalytic properties of differently oriented (hkl) domains of polycrystalline samples or even to obtain the component-specific kinetic data on a microstructured surface with several active components. Applying this approach to different μm-sized Pt(hkl)- and Pd(hkl)-domains in the CO oxidation reaction, specific behavior of particular orientations in the catalytic ignition was revealed. It has also been shown that for the CO oxidation reaction, the typical surface science approach based on isothermal monitoring of kinetic transitions, yields quantitatively the same results as the common technical catalysis approach based on isobaric study of different reaction regimes, at least in the pressure range of 10^− 5^ mbar.

Using the present approach, the role of artificially created defects and steps in the local reaction kinetics of CO oxidation on low-index Pd domains was studied. Again, the exact identity of the experimental conditions for the different regions of the sample and simultaneous collection of local information allowed direct comparison of different orientations. Using a model catalyst consisting of Pd powder agglomerates supported by a polycrystalline Pt foil, local kinetic phase diagrams for CO oxidation on the selected (100) and (110) domains of the Pt foil and for the different micrometer-sized Pd powder agglomerates were obtained. The present study on individual μm-sized domains of polycrystalline samples and on a two-component (Pt/Pd) model system was performed using a standard PEEM with a μm-resolution. The ability to resolve the catalytic activity of individual active domains or components is, however, limited in the proposed approach solely by the spatial resolution of the used PEEM, i.e. employing the best modern devices with resolution near the lower nm-range, the local kinetic analysis of individual catalytic particles in a commercial catalyst should be feasible.
